# Parasitism by *Aleiodes ceres* Shimbori, 2023 (Hymenoptera: Braconidae) of three species of *Spodoptera* Guenée, 1852: effects of preferential instar and host diet

**DOI:** 10.1007/s10526-024-10287-w

**Published:** 2024-11-12

**Authors:** Jailma Rodrigues dos Santos, Tamara Akemi Takahashi, Gabriel Rodrigues Palma, Rafael de Andrade Moral, José Roberto Postali Parra

**Affiliations:** 1 Department of Entomology and Acarology, Luiz de Queiroz College of Agriculture (ESALQ), University of São Paulo (USP), Piracicaba, SP Brazil; 2 Department of Mathematics and Statistics, Maynooth University, Maynooth, Co. Kildare Ireland; 3 Hamilton Institute, Maynooth University, Mayooth, Co. Kildare, Ireland

**Keywords:** Larval parasitoid, Rogadinae, Soybean, Corn, Biological control

## Abstract

A recently discovered koinobiont parasitoid species, *Aleiodes ceres* Shimbori, 2023, proved to be a promising biological control agent for larvae of *Spodoptera cosmioides* (Walker, 1858), *S. eridania* (Stoll, 1782), and *S. frugiperda* (J.E. Smith, 1797), all well-known pests that cause critical damage to soybean and corn crops. This study investigated the potential of parasitism by *A. ceres* in these three species, including different instars (1st, 2nd, 3rd, and 4th instar) and diets (artificial and natural). Parasitism was highest when it occurs in the second instar for all three host species. The egg-to-pupa period of *A. ceres* was shorter in the second and third instars in relation to the first instar. Parasitism was substantially enhanced by the use of natural diets (soybean and corn leaves) compared with the artificial diet in all three species of the *Spodoptera* complex, suggesting a possible allelochemical interaction. Based on the results from the three host species, the potential for control of *Spodoptera* spp. by *A. ceres* was evident.

**Supplementary Information:**

The online version contains supplementary material available at 10.1007/s10526-024-10287-w.

## Introduction

The genus *Spodoptera* Guenée, 1852 (Lepidoptera, Noctuidae) comprises 31 species, 15 of which are important agricultural pests in the Western and Eastern hemispheres (Kergoat et al. 2021) and four of these having economic importance in Brazil: *Spodoptera frugiperda* (J.E. Smith, 1797), *Spodoptera cosmioides* (Walker, 1858), *Spodoptera eridania* (Stoll, 1782), and *Spodoptera albula* (Walker, 1857) (Parra and Omoto 2004; Parra et al. 2022). Some of the main pests of cotton (*Gossypium hirsutum* L.), corn or maize (*Zea mays* L.), and soybean (*Glycine max* L.) are part of the *Spodoptera* complex (Parra et al. 2022) and cause significant damage from their voracious attacks (Pogue 2002; Silva et al. 2017; Specht and Roque-Specht 2019).

In Brazil, the corn armyworm, *S. frugiperda*, causes the greatest economic losses. This pest, native to the Americas, is highly polyphagous (Pogue 2002; Early et al. 2018; Adhikari et al. 2020). In 2016, it was recorded in Africa (Goergen et al. 2016), in 2018 in Asia (India) (Ganiger et al. 2018; Sharanabasappa et al. 2018), and in 2020 in Oceania (Australia) (EPPO 2020). Recently, traditional control methods have not yielded satisfactory results, because of the armyworm’s resistance to different chemical groups and to transgenic corn expressing toxins from Bt (*Bacillus thuringiensis* Berliner) (Cruz et al. 2012; van den Berg and du Plessis 2022; APRD 2023).

Both *S. cosmioides* and *S. eridania* have also become more concerning since the 2013–2014 harvest, with population outbreaks in genetically modified soybean that expresses the Cry1Ac protein. Because of the low susceptibility of *Spodoptera* complex larvae to Bt proteins (CTNBio 2010; Bernardi et al. 2014), these two species are currently the main defoliating pests of soybean.

As a result of the inefficiency of chemical insecticides and/or Bt transgenic crops in controlling members of the *Spodoptera* complex, it is essential to investigate alternative individual or combined strategies to reduce losses from attacks by these pests, based on Integrated Pest Management (IPM) principles. Biological control with parasitoids is a long-lasting and environmentally safe option.

Investigations of the use of biological control to manage *Spodoptera* species, mainly *S. frugiperda,* are being conducted with the egg parasitoids *Telenomus remus* Nixon, 1937 (Hymenoptera: Scelionidae) and species of *Trichogramma* Westwood, 1833 (Hymenoptera: Trichogrammatidae) (Goulart et al. 2011; Wengrat et al. 2021; Fortes et al. 2023). As the results of these attempts have not so far been satisfactory, it is necessary to consider combinations of species or other natural enemies that are simpler to rear and easily adapt to different climatic regions.

Recently, Shimbori et al. (2023) collected and described a new species of parasitoid wasp, *Aleiodes ceres* Shimbori, 2023 (Hymenoptera: Braconidae), and suggested that it might be a useful biological control agent for the *Spodoptera* complex. *Aleiodes ceres* is a solitary koinobiont endoparasitoid (Shaw 2006; Abreu et al. 2014) and was first collected in corn and soybean fields from early instar larvae of *S. cosmioides, S. eridania*, and *S. frugiperda* in a temperate region of Brazil, São José dos Pinhais, state of Paraná (Shimbori et al. 2023).

This study aimed to identify the instars of the hosts *S. frugiperda*, *S. eridania* and *S. cosmioides* that support the highest parasitism of *A. ceres*. Additionally, we evaluated the effect of different diets offered to the hosts on the success of *A. ceres* parasitism.

## Materials and methods

### Insect rearing

The hosts and the parasitoid were reared in the Insect Biology Laboratory, Entomology and Acarology Department, University of São Paulo (USP)/Luiz de Queiroz College of Agriculture (ESALQ), Piracicaba, SP, Brazil. The insects were kept under controlled conditions (25 ± 2 °C, 70 ± 10% RH, and a L:D 14:10 photoperiod). The hosts *S. cosmioides*, *S. eridania*, and *S. frugiperda* were reared on artificial diets as proposed by Greene et al. (1976), following the rearing method described by Parra (2015).

Adults of *A. ceres* were kept in cages (30 × 27 × 25 cm) covered with anti-aphid screening (0.87 mm) and were provided with water in moistened cotton rolls and fed droplets of pure honey. Three cages of *A. ceres* were maintained, one for each species of *Spodoptera*. The larvae offered for parasitism were from the laboratory rearing colony of each *Spodoptera* species, fed the above-mentioned artificial diet.

Maintenance of the parasitoid cages was carried out every 48 h. The parasitized hosts were kept in transparent polyethylene pots (200 ml) containing an artificial diet for the larvae, since the parasitoid is a koinobiont. After the larval stage, the parasitoid pupae were transferred to 50 ml pots until emergence, then transferred to their respective rearing cages.

For all the experiments, the cages for the adults were made of 500 ml plastic pots inverted over the lids, containing pure honey for the parasitoids as well as artificial and/or natural diets for the host larvae (particular to each experiment). The upper part of each pot was pierced to allow air circulation.

### Bioassay 1: Parasitism and development of *A. ceres* in different instars of three *Spodoptera* species

An experiment was set up using a completely randomized design (CRD) with 12 treatments in a factorial scheme, and 20 replicates. The two factors were host species (with three levels: *S. cosmioides*, *S. eridania*, and *S. frugiperda*) and larval instar (with four levels: 1st, 2nd, 3rd, and 4th instar, identified by head-capsule measurement). Each replicate consisted of a pair of newly emerged parasitoids alongside 15 larvae offered to be parasitized. The number of larvae provided was determined based on the limitation of daily parasitism, allowing for an average of eight ovipositions per day (van Achterberg and Shaw 2016). The sexes were identified by inspecting the presence of an ovipositor in the female.

For mating, newly emerged males and females were kept in flat-bottom glass tubes (1.5 × 6 cm), containing droplets of pure honey and sealed with transparent plastic. These couples were kept in air-conditioned chambers set at 25 ± 2 °C; 70 ± 10% RH, and a L:D 14:10 photoperiod. After this period, the couples were transferred to the cages described above, containing larvae of each instar of the *Spodoptera* species to be tested, fed on the artificial diet.

The host larvae were offered to be parasitized for 24 h. After this period they were removed and placed in 200 ml pots containing the same artificial diet. To limit possible cannibalism, especially by *S. frugiperda*, unparasitized larvae were removed each day. Unparasitized larvae were separated and judged by the size and development time in comparison with parasitized larvae. The following variables were recorded: (1) percentage of parasitism; (2) duration (days) of the egg-to-pupa (“mummification” of the host), pupa-to-adult, and egg-to-adult periods; (3) percentage of emergence of parasitoids (relationship between the number of successfully parasitized caterpillars and the total number of emerged adults); and (4) sex ratio (proportion females) of *A. ceres*. For this last variable, replicates that generated only male progeny were excluded.

### *Aleiodes ceres* parasitism on three species of *Spodoptera* fed artificial and natural diets

#### Bioassay 2: Artificial diet vs. soybean leaves

Second-instar larvae (judged to be the optimal instar from the results of bioassay 1) of *S. cosmioides*, *S. eridania*, and *S. frugiperda*, reared on artificial diet, were exposed for parasitism by *A. ceres*. The first treatment consisted of larvae fed the artificial diet from Greene et al. (1976). In the second treatment, the larvae were fed soybean leaves.

These treatments were used to evaluate if soybean leaves (natural diet) would be a more efficient attractant, thus optimizing parasitism by *A. ceres*. The design was completely randomized (CRD), following a 3 × 2 factorial scheme, which corresponded to the three host species (*S. cosmioides, S. eridania*, and *S. frugiperda*) and the two types of diets (artificial and natural), totaling six treatments, with 20 replicates for each treatment. A method similar to bioassay 1 was used.

For the natural-diet treatments, the larvae were allowed to feed on the soybean leaves (IAC Foscarin variety) for 10 min, since they were previously fed an artificial diet. A screen was placed inside the cage to support the leaf and facilitate the movement of the parasitoids. For both treatments, the parasitism exposure period was 24 h. After the host larvae were exposed to parasitism, the procedures and evaluations were similar to bioassay 1.

During this experiment, the behavior of *A. ceres* was also investigated, including the foraging time (period needed for the parasitoid to locate the host), host acceptance rate (proportion of samples in which parasitism behavior, i.e., host-parasitoid interaction, was observed), and time of parasitism (from acceptance of the host to removal of the ovipositor), in 15 replicates for each treatment. These parameters were observed for 20 min, beginning 10 min after the larvae were offered the soybean leaves. The following variables were recorded: (1) foraging time (min), (2) host acceptance rate (%), (3) time of parasitism (min), and (4) parasitism (%) of *A. ceres*.

#### Bioassay 3: Artificial diet vs. corn and soybean leaves for *S. frugiperda*

Considering the preference of *S. frugiperda* to feed on corn leaves (AI Paraguaçu variety), this bioassay aimed to determine whether there was any difference in the parasitism of *A. ceres* in larvae of this host species. Thus, larvae fed corn leaves were compared with those fed an artificial diet and soybean leaves.

An experiment was set up using a completely randomized design (CRD), with three treatments related to artificial, corn-leaf, and soybean-leaf diets, including 20 replicates. The procedure and variables were similar to those in bioassay 2.

### Statistical analysis

The percentage of parasitism was analyzed by fitting beta-binomial generalized additive models for location, scale, and shape (GAMLSS) (Stasinopoulos et al. 2017), including the effects of species, instars, and interaction between species-instars on the linear predictors for mean (logit link) and dispersion (log link) for bioassay 1. For bioassay 2, the effects of species, diet, and the interaction between species were included in the linear predictors. Finally, for bioassay 3, only the effect of diet was included in the linear predictors. The sex ratio was analyzed by fitting a beta-binomial GAMLSS with the same linear predictors for bioassay 1.

The time-until-event variables (durations of egg-to-pupa, pupa-to-adult, and egg-to-adult periods) were analyzed by fitting Cox proportional-hazards models, including the effects of species, instars, interaction between species and instars, and a gamma frailty to account for correlations between observations taken within the same experimental unit in the linear predictor (log link). Additionally, the proportion of parasitoid emergence was analyzed by fitting a binomial Generalized Linear Model (GLM) (McCullagh and Nelder 1989; Demétrio et al. 2014), including the effects of species, instars, and interaction between species-instars in the linear predictor (logit link) for bioassay 1.

The foraging time was analyzed by fitting a Weibull GAMLSS, including the effect of species, diet, and interaction between species and diet in the linear predictors for the mean and dispersion (both log links), for bioassay 2. For bioassay 3, only the diet effect was included in the linear predictors. The time of parasitism was analyzed by fitting a gamma GAMLSS, including the effects of species, diet, and interaction between species and diet on the linear predictors for the mean and dispersion (both log links), for bioassay 2. For bioassay 3, the previous variable was analyzed by fitting an inverse gamma GAMLSS, including the effect of diet on the linear predictors for the mean and dispersion (both log links). Host acceptance was analyzed by fitting a binomial GLM, with the same effects included in the linear predictors as specified above, for each biossay.

The significance of the effects, including multiple interactions and isolated effects, was assessed through likelihood ratio (LR) tests for nested models. goodness-of-fit for GAMLSS was assessed through worm-plots (Stasinopoulos et al. 2017), for GLMs using half-normal plots with a simulated envelope (Moral et al. 2017). For Cox proportional hazards models, the goodness-of-fit was assessed by visual analysis of the martingale residuals (Therneau and Grambsch 2000). All analyses were carried out using R (R Core Team 2024).

## Results

### Bioassay 1: Parasitism and development of *A. ceres* in different instars of *Spodoptera* spp.

**Fig. 1:**
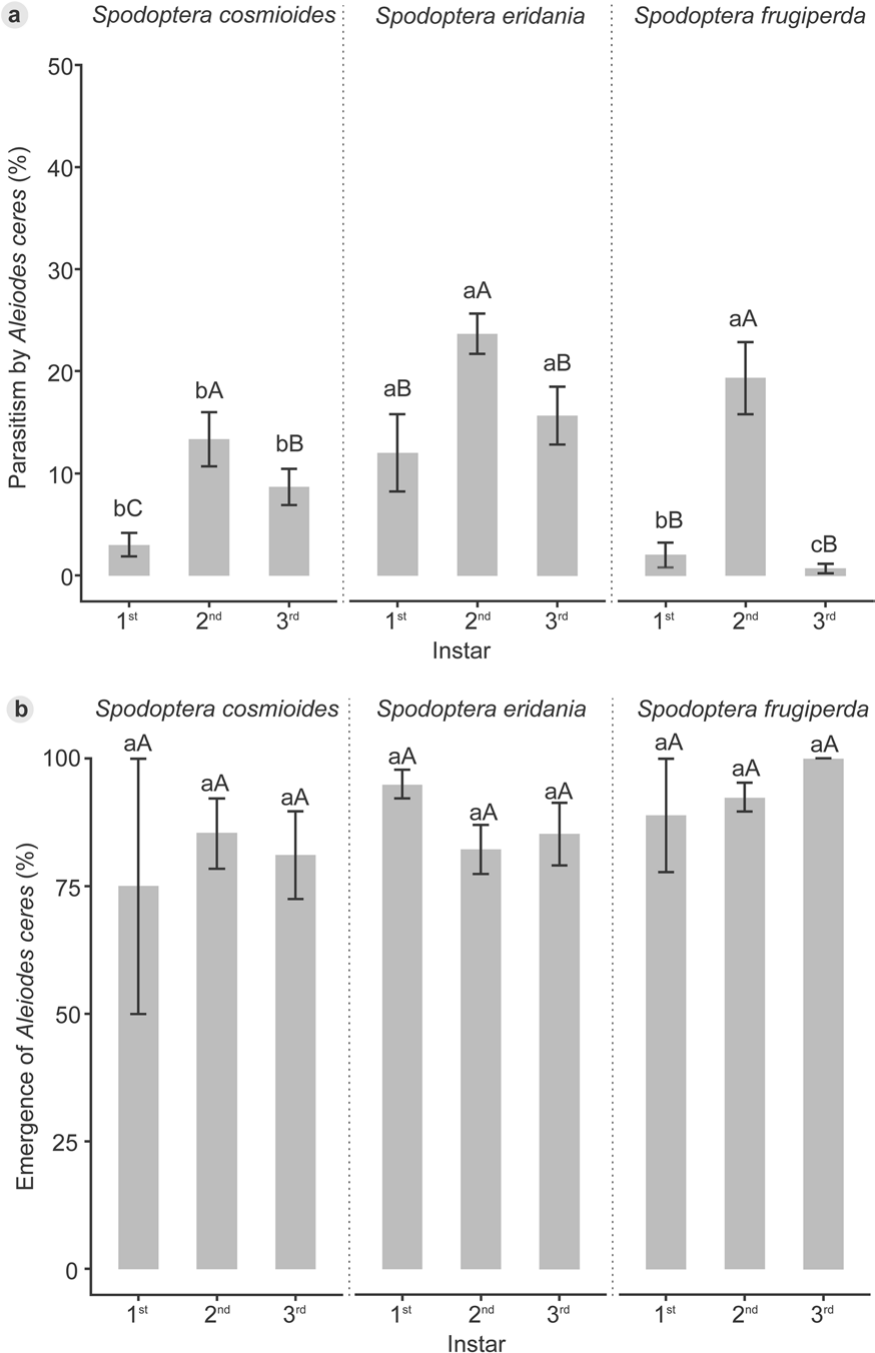
Mean parasitism (%) (a) and emergence (%) (b) of *Aleiodes ceres* ± SE in different instars of three species of the *Spodoptera* complex at 25 ± 2 °C, 70 ± 10% RH, and a L:D 14:10 photoperiod. Lowercase letters represent differences among species. Uppercase letters represent differences among instars (5% significance level)

Analysis of the parasitism data revealed a significant interaction between species and instars (χ^2^ = 26.53; df = 4; *p* < 0.001), indicating that *A. ceres* showed greatest fitness when it parasitized second-instar larvae, in comparison to the first and third instars of all three species. The highest percentages of parasitism were observed in the second-instar larvae of S. *eridania* (23.70 ± 1.96%) and *S. frugiperda* (19.30 ± 3.52%), compared to *S. cosmioides* (13.30 ± 2.65%). Additionally, *S. eridania* demonstrated higher parasitism percentages in the first (12.00 ± 3.77%) and third (15.70 ± 2.83%) larval instars when compared to both *S. cosmioides* (3.00 ± 1.13% and 8.67 ± 1.75%) and *S. frugiperda* (2.00 ± 1.19% and 0.67 ± 0.46%). Fourth-instar larvae were not parasitized. Therefore, this stage was not included in the statistical analysis since it did not contribute to the variability (Fig. 1a, Supplementary Table S1).

The duration of the egg-to-pupa period of *A. ceres* differed among the three species (χ^2^= 76.55; df = 2; *p* < 0.001), with the shortest times observed for *S. frugiperda* (ranging from 8.62 ± 0.09 to 9.00 ± 1.00 days), followed by *S. eridania* (8.73 ± 0.16 to 11.00 ± 0.17 days) and *S. cosmioides* (10.2 ± 0.42 to 13.20 ± 0.70 days)*.* In the second (ranging from 8.62 ± 0.09 to 11.10 ± 0.45 days) and third (8.73 ± 0.16 to 10.20 ± 0.42 days) instars, *A. ceres* developed more rapidly during the egg-to-pupa period in comparison with the first larval instar (9.33 ± 0.21 to 13.20 ± 0.70 days) (χ^2^ = 89.76, df = 2, *p* < 0.001). There was also a difference for the egg-adult period, but it was significant only for the species factor (χ^2^ = 29.39; df = 2; *p* < 0.001), with *A. ceres* developing in less time in the hosts *S. frugiperda* (14.80 ± 1.25 to 16.00 ± 1.00 days) and *S. eridania* (15.40 ± 0.49 to 17.80 ± 0.29 days), while the development time in the host *S. cosmioides* tended to increase in this period (16.20 ± 1.13 to 18.30 ± 0.28 days)*.* There was no difference among the three hosts for the pupa-to-adult period (χ^2^= 2.33, df = 2, *p* = 0.31), ranging from 7.00 ± 0.00 to 7.81 ± 0.23 days (Table 1, Supplementary Figure S1, Supplementary Table S1).

**Table 1:** Mean development time of egg-to-pupa, pupa-to-adult, and egg-to-adult periods (days) of *Aleiodes ceres* in different instars of three species of the *Spodoptera* complex at 25 ± 2 °C, 70 ± 10% RH, and a L:D 14:10 photoperiod

Species	Instars			
	1st	2nd	3rd	4th*
	Egg-to-pupa (days) ± SE			
*Spodoptera cosmioides*	13.20 ± 0.70 ^cB^	11.10 ± 0.45 ^cA^	10.20 ± 0.42 ^cA^	–
*Spodoptera eridania*	11.00 ± 0.17 ^bB^	9.01 ± 0.09 ^bA^	8.73 ± 0.16 ^bA^	–
*Spodoptera frugiperda*	9.33 ± 0.21 ^aB^	8.62 ± 0.09 ^aA^	9.00 ± 1.00 ^aA^	–
	Pupa-to-adult (days) ± SE			
*Spodoptera cosmioides*	7.50 ± 0.86 ^aA^	7.40 ± 0.15 ^aA^	7.69 ± 0.36 ^aA^	–
*Spodoptera eridania*	7.19 ± 0.32 ^aA^	7.24 ± 0.18 ^aA^	7.81 ± 0.23 ^aA^	–
*Spodoptera frugiperda*	7.75 ± 1.03 ^aA^	7.61 ± 0.31 ^aA^	7.00 ± 0.00 ^aA^	–
	Egg-to-adult (days) ± SE			
*Spodoptera cosmioides*	16.20 ± 1.13 ^bA^	18.30 ± 0.28 ^cA^	16.30 ± 0.50 ^cA^	–
*Spodoptera eridania*	17.80 ± 0.29 ^cA^	16.60 ± 0.16 ^bA^	15.40 ± 0.49 ^aA^	–
*Spodoptera frugiperda*	14.80 ± 1.25 ^aA^	15.80 ± 0.26 ^aA^	16.00 ± 1.00 ^bA^	–

^*^Fourth-instar larvae were not parasitized. Lowercase letters represent differences among species. Uppercase letters represent differences among instars (5% significance level)

The emergence of *A. ceres* varied from 75.00 ± 25.00% to 100.00 ± 0.00% and the interaction between species and instars was not significant (χ^2^= 2.21; df = 4; *p* = 0.70) (Fig. 1b, Supplementary Table S1). Also, there were no differences among species (χ^2^= 4.00, df = 2, *p* = 0.13) and instars (χ^2^ = 1.42, df = 2, *p* = 0.49). For the three species, there was no significant interaction between species and instars in the sex ratio (χ^2^= 5.17, df = 4, *p* = 0.27) and no difference among species (χ^2^= 2.36, df = 2, *p* = 0.31) and instars (χ^2^= 4.28, df = 2, *p* = 0.12) for this variable, which ranged from 0.68 ± 0.07 to 1.00 ± 0.00 (Table 2, Supplementary Table S1).

**Table 2:** Mean sex ratio (proportion females ± SE) of *Aleiodes ceres* in different instars of three species of the *Spodoptera* complex at 25 ± 2 °C, 70 ± 10% RH, and a L:D 14:10 photo

Species	Instars			
	1st	2nd	3rd	4th*
*Spodoptera cosmioides*	1.00 ± 0.00^aA^	0.83 ± 0.06^aA^	1.00 ± 0.00^aA^	–
*Spodoptera eridania*	0.77 ± 0.12^aA^	0.68 ± 0.07^aA^	0.82 ± 0.07^aA^	–
*Spodoptera frugiperda*	1.00 ± 0.00^aA^	0.77 ± 0.05^aA^	1.00 ± 0.00^aA^	–

*Fourth-instar larvae were not parasitized

Lowercase letters represent differences among species. Uppercase letters represent differences among instars (5% significance level)

### *Aleiodes ceres *parasitism on three species of *Spodoptera* fed artificial and natural diets

#### Bioassay 2: Artificial diet vs. soybean leaves

The foraging time differed significantly in regard to the species factor (χ^2^= 7.11; df = 2; *p* = 0.03) and the diet factor (χ^2^= 17.76; df = 1; *p* < 0.001). With the artificial diet, the *A. ceres* foraging time was shortest for *S. eridania* (7.14 ± 1.81 min); while with the natural diet, the foraging time was shortest for *S. frugiperda* (3.32 ± 0.42 min), followed by *S. eridania* (4.10 ± 0.63 min)*.* Soybean leaves favored shorter foraging times, ranging from 3.32 ± 0.42 to 5.75 ± 0.77 min (Table 3, Supplementary Table S2).

**Table 3:** Mean foraging time (min), host acceptance rate (%), and parasitism time (min) of *Aleiodes ceres* in three species of the *Spodoptera* complex, fed different diets [artificial and natural (soybean leaves)] at 25 ± 2 °C, 70 ± 10% RH, and a L:D 14:10 photoperiod

Species	Diets	
	Artificial	Natural
	Foraging time (min) ± SE	
*Spodoptera cosmioides*	8.07 ± 1.90^abB^	5.75 ± 0.77^bA^
*Spodoptera eridania*	7.14 ± 1.81^aB^	4.10 ± 0.63^abA^
*Spodoptera frugiperda*	8.50 ± 0.33^bB^	3.32 ± 0.42^aA^
	Host acceptance (%) ± SE	
*Spodoptera cosmioides*	33.00 ± 13.00^aB^	100.00 ± 0.00^aA^
*Spodoptera eridania*	47.00 ± 13.00^aB^	100.00 ± 0.00^aA^
*Spodoptera frugiperda*	33.00 ± 13.00^aB^	87.00 ± 9.00^aA^
	Parasitism time (min) ± SE	
*Spodoptera cosmioides*	3.85 ± 0.49^bB^	2.81 ± 0.25^bA^
*Spodoptera eridania*	1.89 ± 0.33^aA^	2.04 ± 0.19^aA^
*Spodoptera frugiperda*	4.67 ± 0.31^bB^	2.17 ± 0.23^abA^

Lowercase letters represent differences among species. Uppercase letters represent differences between diets (5% significance level)

The host acceptance rate of *A. ceres* differed only for the diet factor (χ^2^= 39.28; df = 1; *p* < 0.001). For hosts offered the artificial diet, the rate varied from 33.00 ± 13.00% to 47.00 ± 13.00% among the species, whereas the acceptance rate for hosts on the natural diet was significantly higher, 87.00 ± 9.00% to 100.00 ± 0.00%, i.e., with a stronger interaction between the host and the parasitoid (Table 3, Supplementary Table S2). Feeding the hosts on soybean leaves might favor parasitism through probable allelochemical interactions.

The parasitism time differed with the diet offered to the host species (χ^2^= 10.63; df = 2; *p* < 0.001). *Aleiodes ceres* responded most rapidly to *S. eridania* fed both diets, artificial (1.89 ± 0.33 min) and natural (2.04 ± 0.19 min), followed by *S. frugiperda* fed soybean leaves (2.17 ± 0.23 min) (Table 3, Supplementary Table S2).

**Fig. 2:**
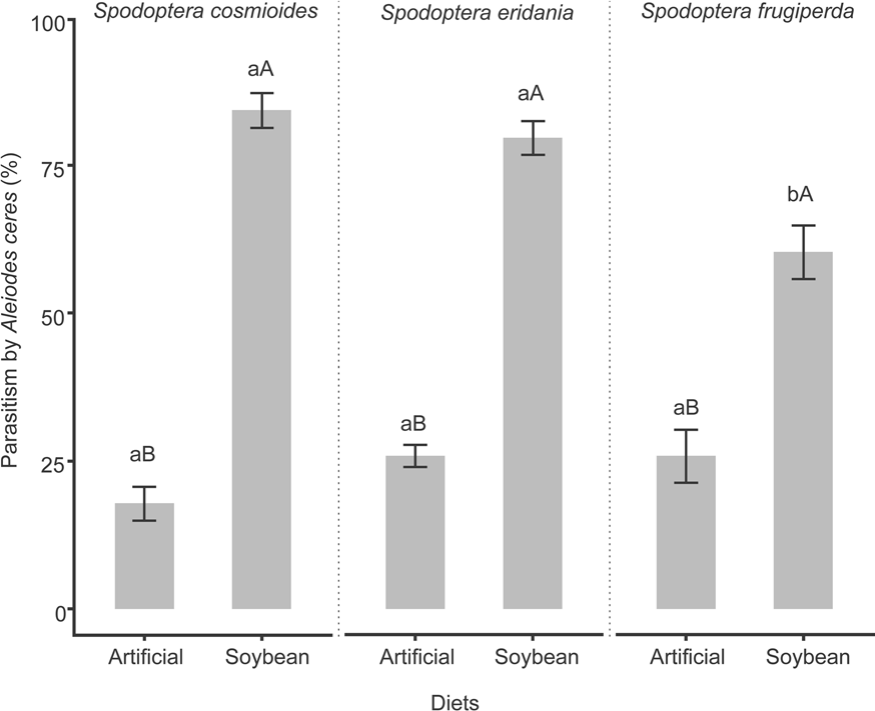
Mean parasitism (%) ± SE of *Aleiodes ceres* in three species of the *Spodoptera* complex fed different diets [artificial and natural (soybean leaves)] at 25 ± 2 °C, 70 ± 10% RH, and a L:D 14:10 photoperiod. Lowercase letters represent differences among the species. Uppercase letters differences between the diets (5% significance level)

The percentage of parasitism showed a significant interaction among the species and diets (χ^2^= 17.46; df = 2; *p* < 0.001). The results indicated that *A. ceres* more actively parasitized *S. cosmioides* and *S. eridania* when these were fed the natural diet during the parasitism, with parasitism percentages of 79.70 ± 2.84% and 84.30 ± 2.95%, respectively, compared to *S. frugiperda* (60.00 ± 0.04%). On the other hand, when fed the artificial diet, all three species showed similar parasitism, ranging from 18.10 ± 2.89% to 26.30 ± 1.90% (Fig. 2, Supplementary Table S2).

#### Bioassay 3: Artificial diet vs. corn and soybean leaves for *S. frugiperda*

The soybean diet resulted in a shorter foraging time by *A. ceres* (χ^2^= 22.50; df = 2; *p* < 0.001), with a mean of 3.32 ± 0.42 min. Acceptance by *A. ceres* was higher when the hosts were fed corn and soybean leaves (χ^2^= 13.14; df = 2; *p* = 0.0014), with 87.00 ± 9.00% of the samples showing host-parasitoid interaction behaviors. The time of parasitism was similar in hosts fed soybean and corn (χ^2^= 18.60; df = 2; *p* < 0.001), with a mean of approximately 2.00 min (Table 4, Supplementary Table S3).

**Table 4:** Mean foraging time (min), frequency of host acceptance (%), and parasitism time (min) of *Aleiodes ceres* in *Spodoptera frugiperda* fed different diets [artificial and natural (corn and soybean)] at 25 ± 2 °C, 70 ± 10% RH, and a 14:10 photoperiod

Diets	Foraging time (min) ± SE	Host acceptance (%) ± SE	Parasitism time (min) ± SE
Artificial	8.50 ± 0.33c	33.00 ± 13.00b	4.67 ± 0.30b
Corn	5.91 ± 0.91b	87.00 ± 9.00a	2.73 ± 0.34a
Soybean	3.32 ± 0.42a	87.00 ± 9.00a	2.17 ± 0.30a

Different letters in the column indicate significant differences among diets (5% significance level)

**Fig. 3:**
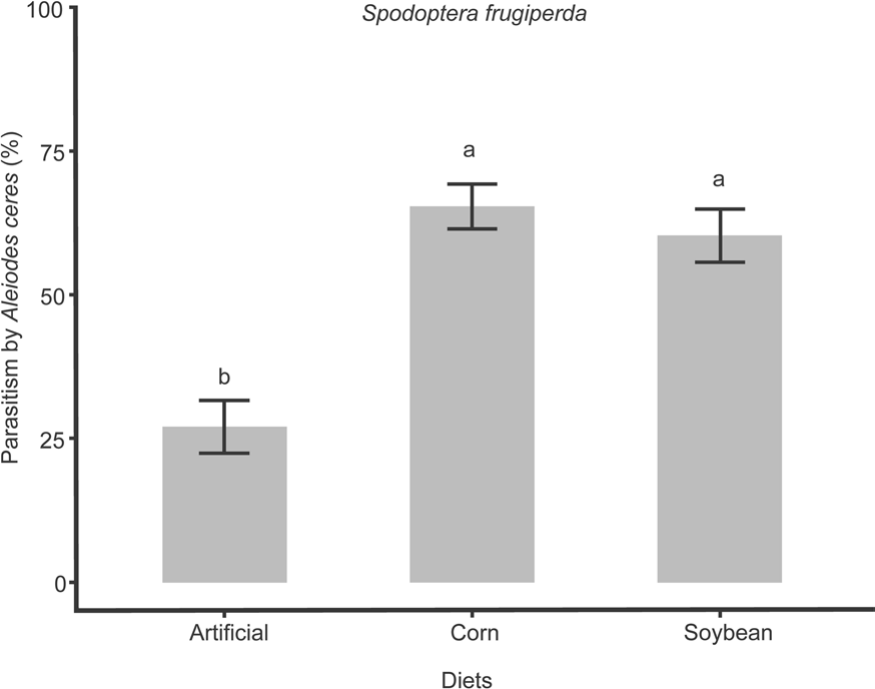
Mean parasitism (%) ± SE of *Aleiodes ceres* in *Spodoptera frugiperda* fed different diets [artificial and natural (corn and soybean)] at 25 ± 2 °C, 70 ± 10% RH, and a L:D 14:10 photoperiod. Different letters indicate significant differences between the diets (5% significance level)

The amount of parasitism corresponds to the behavior of *A. ceres* and showed higher percentages in hosts fed natural diets (χ^2^= 39.03; df = 2; *p* < 0.001). It reached 65.30 ± 3.86% in corn and 60.30 ± 4.57% in soybean, approximating a mean of ten and nine parasitized larvae respectively (Fig. 3, Supplementary table S3).

## Discussion

The results from this investigation demonstrated the potential of biological control of *A. ceres* in the laboratory. However, it is necessary to conduct semi-field and field studies and to develop mass-rearing production methods before *A. ceres* could be used in biological control programs to control the *Spodoptera* complex in Brazil. Currently, only the egg parasitoid *Trichogramma pretiosum* Riley, 1879 (Hymenoptera: Trichogrammatidae) is used against *S. frugiperda* in corn, and rarely against *S. eridania* in cotton crops.

Because *A. ceres* was recently described (Shimbori et al. 2023), little is known about its biology, although some information is available for other species of *Aleiodes*. Given the importance of appropriate host selection for success in the use of a parasitoid for biological control (Mattiacci and Dicke 1995), this study presents new data on the biology and behavior of *A. ceres* parasitizing *S. cosmioides, S. eridania*, and *S. frugiperda*.

To rear *A. ceres*, it must be provided with hosts in the second instar. This contrasts with some other braconids used in biocontrol, such as *Cotesia flavipes* (Cameron, 1891) (Hymenoptera: Braconidae), which rely on more advanced instars and are reared under laboratory conditions for release on three to four million hectares of sugarcane to control *Diatraea saccharalis* (Fabricius, 1794) (Lepidoptera: Crambidae) (Cherubin 2018; Pinto and Trujillo 2019; Pinto 2021)*.* Other braconid species prefer the initial instars of *S. frugiperda*, such as *Cotesia icipe* Fernández-Triana & Fiaboe, 2017 (Hymenoptera: Braconidae) (Mohamed et al. 2021) and *Microplitis manila* (Ashmead, 1904) (Hymenoptera: Braconidae) (Xing et al. 2022).

Considering the parasitization characteristics of *A. ceres* described here, it would be possible to mass-rear this species on the fall armyworm, since cannibalism behavior occurs only after the third-instar larva (Tang et al. 2022). The insects could be reared in groups in a mass system, with the precaution that second instar larvae would be used to avoid the risk of cannibalism.

Although *A. ceres* was collected in a temperate region, the parasitoid efficiently parasitized all three species, which occur under different climatic conditions in Brazil. The thermal requirements of the parasitoid need to be studied. The requirements of the members of the *Spodoptera* complex investigated here have been evaluated previously (Parra et al. 2022).

The possibility that the natural diet for species of the *Spodoptera* complex could attract and optimize the activity of *A. ceres* was confirmed by observation of the foraging time, host acceptance rate, and percentage of parasitism. For all three host species, the diet of soybean leaves, as well as corn leaves for *S. frugiperda*, contributed to foraging rapidity, a higher acceptance rate, and an increase in *A. ceres* parasitism, suggesting the involvement of volatiles released by the leaves of these crops.

The presence of allelochemicals has been demonstrated by several authors, acting on different parasitoid species in tritrophic interactions (Silveira et al. 2018; Turlings and Erb 2018; Riffel et al. 2021; Souza et al. 2022). One alternative to increase the feasibility of rearing the parasitoids on artificial diet would be to evaluate the leaf volatiles as food additives, or to produce extracts from the plants and mix them into the diets, similar to the procedure used at the beginning of development of the artificial diet, especially by French researchers (J.R.P. Parra, personal information). Nevertheless, it is possible that, with the passage of generations in the laboratory, insects may become increasingly acclimated to the artificial diet (without plants), which may be reflected in an increase in parasitism.

The emergence of *A. ceres* was higher than 75% under all experimental conditions, showing that the parasitoid can develop well in these members of the *Spodoptera* complex. In general, there is no evidence of sex ratio modification from the use of an artificial diet, confirming the possibility of rearing *A. ceres* with this food, without altering the feasibility of use and population increase of the parasitoid under laboratory conditions.

This study demonstrated that *A. ceres* can effectively control the hosts *S. cosmioides, S. eridania,* and *S. frugiperda* during their early developmental stages, with the second instar being the most suitable for parasitism. The natural diet is crucial for attracting *A. ceres*, while the best hosts for rearing on an artificial diet are *S. eridania*, followed by *S. frugiperda* and *S. cosmioides*.

## Supplementary Information

Below is the link to the electronic supplementary material.

Supplementary file1 (DOCX 214 KB)
