# Supplementary material for: Parasitism by Aleiodes ceres Shimbori, 2023 (Hymenoptera: Braconidae) of three species of Spodoptera Guenée, 1852: effects of preferential instar and host diet
Source: Biocontrol (Dordr). 2024 Nov 12;70(1):45–55. doi: 10.1007/s10526-024-10287-w (PMC11772551; doi:10.1007/s10526-024-10287-w)
Supplement: Supplementary file 1 — Supplementary file1 (DOCX 214 KB) [file 10526_2024_10287_MOESM1_ESM.docx]

**Biocontrol**

**Parasitism by *Aleiodes ceres* Shimbori, 2023 (Hymenoptera: Braconidae) of three species of *Spodoptera* Guenée, 1852: Effects of preferential instar and host diet**

**Supplementary Material**

**Fig. S1** Life cycle stages of *Aleiodes ceres*


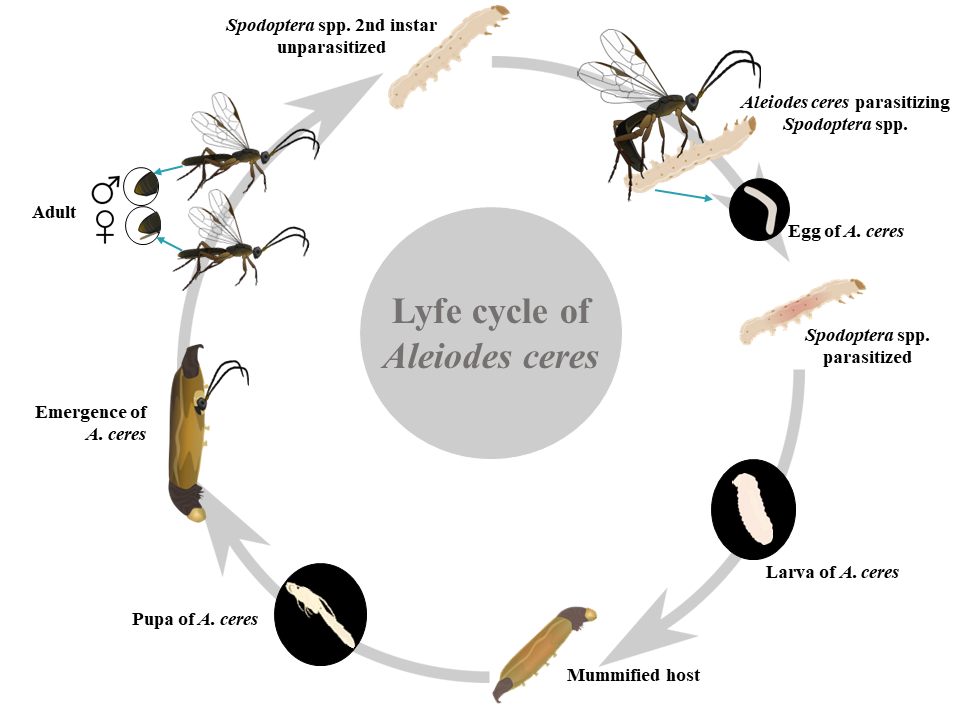


**Table S1.** Statistical data for the variables investigated during the study of the parasitism and development of *Aleiodes ceres* in different instars of three *Spodoptera* species.

| **Variables** | **Model** | **Distribution** | **Link function** | **Factors** | $\chi^{2}$ | **Df** | **P (z)** |
| --- | --- | --- | --- | --- | --- | --- | --- |
| Parasitism (%) | GAMLSS | Beta-binomial | Mean (Logit)  Dispersion (Log) | Species* | 23.69 | 2 | < 0.001 |
|  |  |  |  | Instars** | 31.57 | 2 | < 0.001 |
|  |  |  |  | Interaction | 26.53 | 4 | < 0.001 |
| Development of egg-to-pupa (days) | Cox proportional hazard | Frailty Gamma | Log | Species | 76.55 | 2 | < 0.001 |
|  |  |  |  | Instars | 89.76 | 2 | < 0.001 |
|  |  |  |  | Interaction | 4.74 | 4 | 0.32 |
| Development of pupa-to-adult (days) | Cox proportional hazard | Frailty Gamma | Log | Species | 2.33 | 2 | 0.31 |
|  |  |  |  | Instars | 4.60 | 2 | 0.10 |
|  |  |  |  | Interaction | 3.29 | 4 | 0.50 |
| Development of egg-to-adult (days) | Cox proportional hazard | Frailty Gamma | Log | Species | 29.39 | 2 | < 0.001 |
|  |  |  |  | Instars | 5.57 | 2 | 0.06 |
|  |  |  |  | Interaction | 2.48 | 4 | 0.64 |
| Emergence (%) | GAMLSS | Binomial | Logit | Species | 4.00 | 2 | 0.13 |
|  |  |  |  | Instars | 1.49 | 2 | 0.49 |
|  |  |  |  | Interaction | 2.21 | 4 | 0.70 |
| Sex ratio  [No. of females / (No. of females + No. of males)]. | GLM | Binomial | Logit | Species | 2.36 | 2 | 0.31 |
|  |  |  |  | Instars | 4.28 | 2 | 0.12 |
|  |  |  |  | Interaction | 5.17 | 4 | 0.17 |
| $\chi^{2}$ = Chi-square value (likelihood ratio); Df = Degrees of freedom; P (z) = Probability;  *****Species: *Spodoptera cosmioides, Spodoptera eridania*, and *Spodoptera frugiperda;*  **Larval instars: First, second, and third. | | | | | | | |

**Table S2.** Statistical data for the variables investigated during the study of the parasitism and development of *Aleiodes ceres* in different instars of three *Spodoptera* species fed artificial and natural diets: artificial diet vs. soybean leaves.

| **Variables** | | **Model** | **Distribution** | **Link function** | **Factors** | $\chi^{2}$ | **Df** | **P (z)** |
| --- | --- | --- | --- | --- | --- | --- | --- | --- |
| Foraging time (min) | | GALMSS | Weibull | Log | Species* | 7.11 | 2 | 0.03 |
|  |  |  |  |  | Diets** | 17.76 | 1 | < 0.001 |
|  |  |  |  |  | Interaction | 2.21 | 2 | 0.33 |
| Host acceptance (%) | | GLM | Binomial | Log | Species | 1.94 | 2 | 0.38 |
|  |  |  |  |  | Diets | 39.28 | 1 | < 0.001 |
|  |  |  |  |  | Interaction | 3.38 | 2 | 0.18 |
| Parasitism time (min) | | GALMSS | Gamma | Log | Species | 22.82 | 2 | < 0.001 |
|  |  |  |  |  | Diets | 18.53 | 1 | < 0.001 |
|  |  |  |  |  | Interaction | 10.63 | 2 | < 0.001 |
| Parasitism (%) | | GALMSS | Beta-binomial | Mean (Logit)  Dispersion (Log) | Species | 24.62 | 2 | < 0.001 |
|  |  |  |  |  | Diets | 149.85 | 1 | < 0.001 |
|  |  |  |  |  | Interaction | 17.46 | 2 | < 0.001 |
| \| $\chi^{2}$ = Chi-square value (likelihood ratio); Df = Degrees of freedom; P (z) = Probability;  *****Species: *Spodoptera cosmioides, Spodoptera eridania* and *Spodoptera frugiperda;*  **Diets: Artificial and natural (soybean leaves). \| \| --- \| | | | | | | | | |
|  |  | | | | | | | |

**Table S3.** Statistical data for the variables investigated during the study of the parasitism of *Aleiodes ceres* in *Spodoptera* *frugiperda* fed artificial and natural diets: artificial diet vs. natural (corn and soybean leaves).

| **Variables** | **Models** | **Distribution** | **Link function** | $\chi^{2}$ | **Df** | **P (z)** |
| --- | --- | --- | --- | --- | --- | --- |
| Foraging time (min) | GALMSS | Weibull | Log | 22.50 | 2 | < 0.001 |
| Host acceptance (%) | GLM | Binomial | Log | 13.14 | 2 | 0.0014 |
| Parasitism time (min) | GALMSS | Inverse gaussian | Log | 18.60 | 2 | < 0.001 |
| Parasitism (%) | GLM | Binomial | Log | 39.03 | 2 | < 0.001 |
| $\chi^{2}$ = Chi-square value (likelihood ratio); Df = Degrees of freedom; P (z) = Probability. | | | | | | |
